# miR-124 attenuates Japanese encephalitis virus replication by targeting DNM2

**DOI:** 10.1186/s12985-016-0562-y

**Published:** 2016-06-21

**Authors:** Songbai Yang, Yue Pei, Xinyun Li, Shuhong Zhao, Mengjin Zhu, Ayong Zhao

**Affiliations:** College of Animal Science and Technology, Zhejiang A&F University, Lin’an, Zhejiang 311300 China; Key Laboratory of Agricultural Animal Genetics, Breeding and Reproduction, Ministry of Education & College of Animal Science and Technology, Huazhong Agricultural University, Wuhan, 430070 China

**Keywords:** miR-124, JEV, DNM2, PK15, Porcine

## Abstract

**Background:**

Japanese encephalitis virus (JEV) is a mosquito-borne flavivirus that causes acute viral encephalitis in humans. Pigs are important amplifier hosts of JEV. Emerging evidence indicates that host microRNAs (miRNAs) play key roles in modulating viral infection and pathogenesis. However, mechanistic studies delineating the roles of miRNAs in regulating host-JEV interactions remain scarce.

**Results:**

In this study, we demonstrated that miR-124 inhibited JEV replication in porcine kidney epithelial PK15 cells. Furthermore, using bioinformatics tools, we identified dynamin2 (DNM2), a GTPase responsible for vesicle scission, as a target of miR-124. Small interfering RNA (siRNA) depletion studies inicated that dynamin2 was required for efficient JEV replication. We also demonstrated that upregulation of miR-124 expression corresponded to decreased expression of its target, DNM2, in the JEV-infected PK15 cells.

**Conclusions:**

Overall, these results suggest the importance of miR-124 in modulating JEV replication and provide a scientific basis for using cellular miRNAs in anti-JEV therapies.

**Electronic supplementary material:**

The online version of this article (doi:10.1186/s12985-016-0562-y) contains supplementary material, which is available to authorized users.

## Background

Japanese encephalitis virus (JEV) is a mosquito-borne neurotropic virus that belongs to the family flaviviridae. JEV is mostly prevalent in eastern, southeastern and southern Asian countries where three billion people are at risk of contracting the disease [1]. A 2011 review estimated that the annual incidence was 1.8/100,000 and 5.4/100,000 for children 0-14 years old in 24 JEV endemic countries [2]. JEV is transmitted in an enzootic cycle between mosquito vectors and vertebrate hosts; humans contract JEV when bitten by an infected mosquito. Pigs act as amplifying hosts of JEV; therefore, the domestic pig was considered to be a risk factor in the transmission of the disease to humans [3, 4]. JEV is also one of the main causes of infectious reproductive failure in swine, and this has resulted in significant economic losses for the swine industry. The primary symptoms of pigs infected with JEV are fetal abortion and stillbirth in infected sows and aspermia in boars [5, 6]. In order to elucidate the pathogenesis of JEV in pigs, numerous studies on JEV have been conducted in PK-15 cells, a porcine kidney epithelial cell line, which have a similar susceptibility as skin epithelial cells to JEV [7–9]. Therefore, PK-15 cells are a good model in which to evaluate the host response to JEV infection.

MicroRNAs (miRNAs) are small non-coding RNAs of approximately 22 nucleotides in length, which play a crucial role in the post-transcriptional regulation of genes involved in fundamental biological processes, including cell differentiation, proliferation, apoptosis and cell signaling [10, 11]. miRNAs regulate gene expression by guiding the RNA-induced silencing complex (RISC) to partially complementary sites within the 3’ UTR of target mRNAs via their seed region [12]. miRNAs have been shown to regulate the host antiviral response by altering the expression of host genes required for virus replication and antiviral response [13–16] or by directly targeting viral genomes and/or transcripts. [17–20]

Recent studies suggest that miRNAs play a notable role in cellular immune response during Japanese encephalitis virus infection [21–24]. For example, miR-15b and miR-155 were shown to induce an inflammatory response in JEV-infected microglial cells via suppression of RNF125 [21] and SHIP1 [23], respectively, whereas miR-146a was shown to inhibit the immune response against JEV by suppressing the JAK/STAT pathway in mouse monocytes [22]. In addition, miR-29b regulated JEV-induced microglia activation, and miR-155 suppressed JEV replication and negatively modulated innate immune responses in microglial cells [24, 25].

In this study, we investigated the role of host miRNAs in JEV infection of porcine cells. We show that expression of miR-124 is upregulated in response to JEV infection and that this results in the suppression of the target gene, DNM2, which is required for virus replication. Thus, we conclude that upregulation of miR-124 and the subsequent suppression of DNM2 represents a host response aimed at limiting JEV infection. Our study reveals an example of a miRNA that modulates JEV replication and also highlights a host factor that could be used for RNAi-mediated antiviral therapeutic strategies.

## Results

### miR-124 inhibits JEV replication

JEV enters the central nervous system and propagates in the neurons, causing a disruption to the blood–brain barrier that results in acute viral encephalitis in humans [26, 27]. miR-124 is highly expressed in neurons (representing 25 % to 48 % of all brain miRNAs), and it was first cloned from the mouse brain where it was later found to mediate neuronal differentiation [28, 29]. In addition, recent studies showed that miR-124 was differentially expressed in JEV-infected porcine cells [30]. Therefore, miR-124 may play a notable role in JEV infection.

**Fig. 1:**
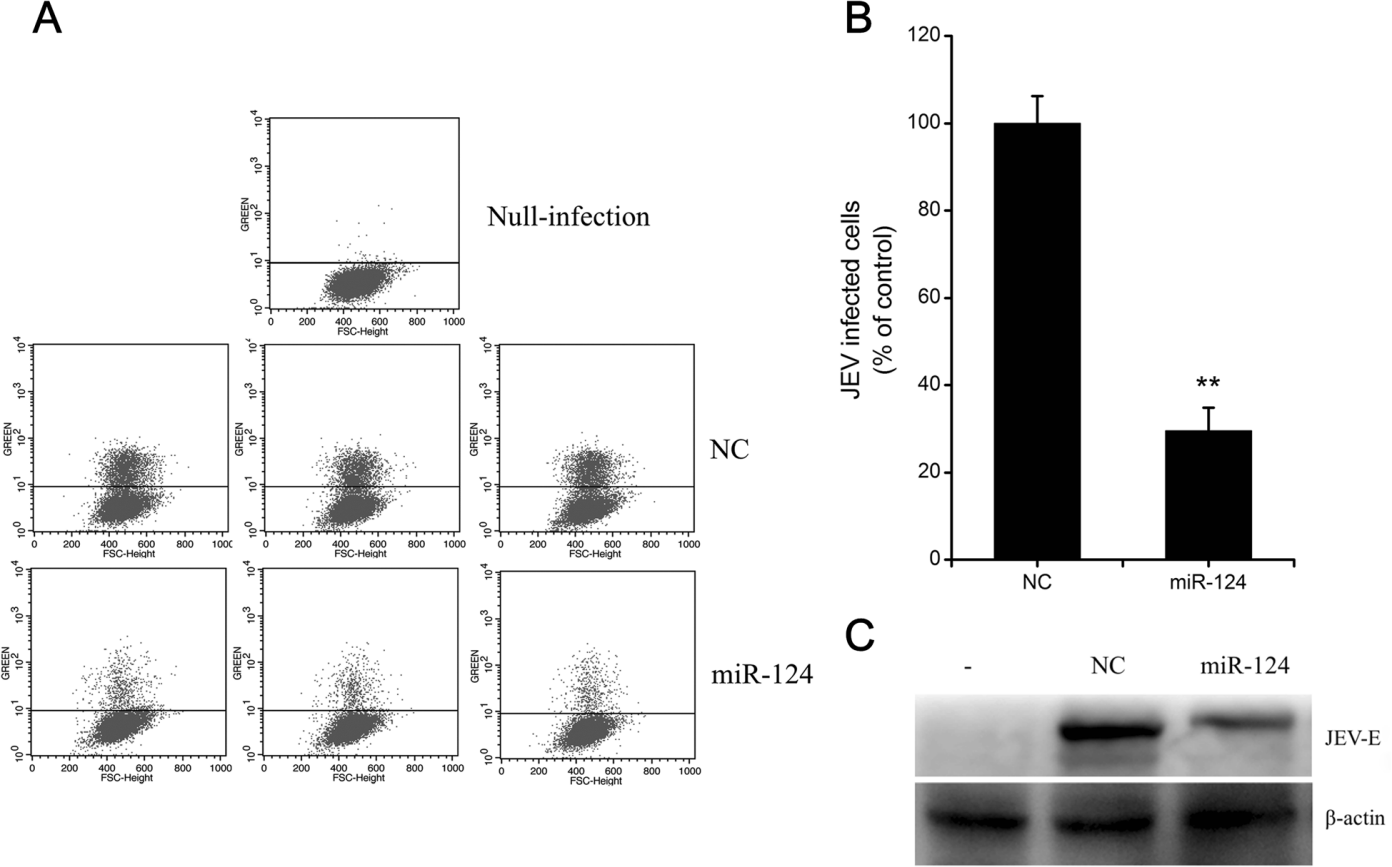
miR-124 attenuates JEV replication in PK15 cells. PK15 cells were transfected with synthetic mimics specific to miR-124 or nonspecific (NC) mimics. At 48 h post-transfection, cells were infected with JEV at MOI = 1. **a** Representative images of flow cytometry analyses for JEV infected cells at 24 hpi. Dots above the solid line represent JEV-infected cells. **b** The percentage of internalized viruses in transfected cells was determined by flow cytometry and normalized to the value for the NC group. The results are presented as the mean ± SD of three independent experiments. **c** At 36 hpi, JEV E protein was analyzed by western blot analysis. β-actin was used as an internal loading control. -, control without JEV infection, **p < 0.01

In order to examine the effect of miR-124 on JEV replication, PK15 cells were transfected with either a miR-124 mimic or a nonspecific (NC) mimic at a concentration of 50 nM, and then were infected with JEV at MOI = 1 after 36 h of transfection. We examined virus production and viral gene expression via flow cytometry assays and western blot analyses. JEV infection was significantly reduced in cells transfected with the miR-124 mimic (Fig. 1A, B), and the expression of JEV E protein was also decreased when compared to cells transfected with the NC mimic (Fig. 1C). The observed changes in the JEV replication efficiency of cells transfected with the miR-124 mimic provide strong evidence for the antiviral activity of miR-124.

### JEV genomic RNA is not a target of miR-124

Host miRNAs were shown to influence the life cycle of RNA viruses by altering host cell gene expression [31, 32]. In addition, many miRNAs have been shown to interact directly with the viral genome RNA to inhibit virus replication [33–35]. In order to determine whether miR-124 inhibits JEV replication via a direct interaction with viral RNA, computational screening of viral RNA for potential miR-124 binding sites was performed.

**Fig. 2:**
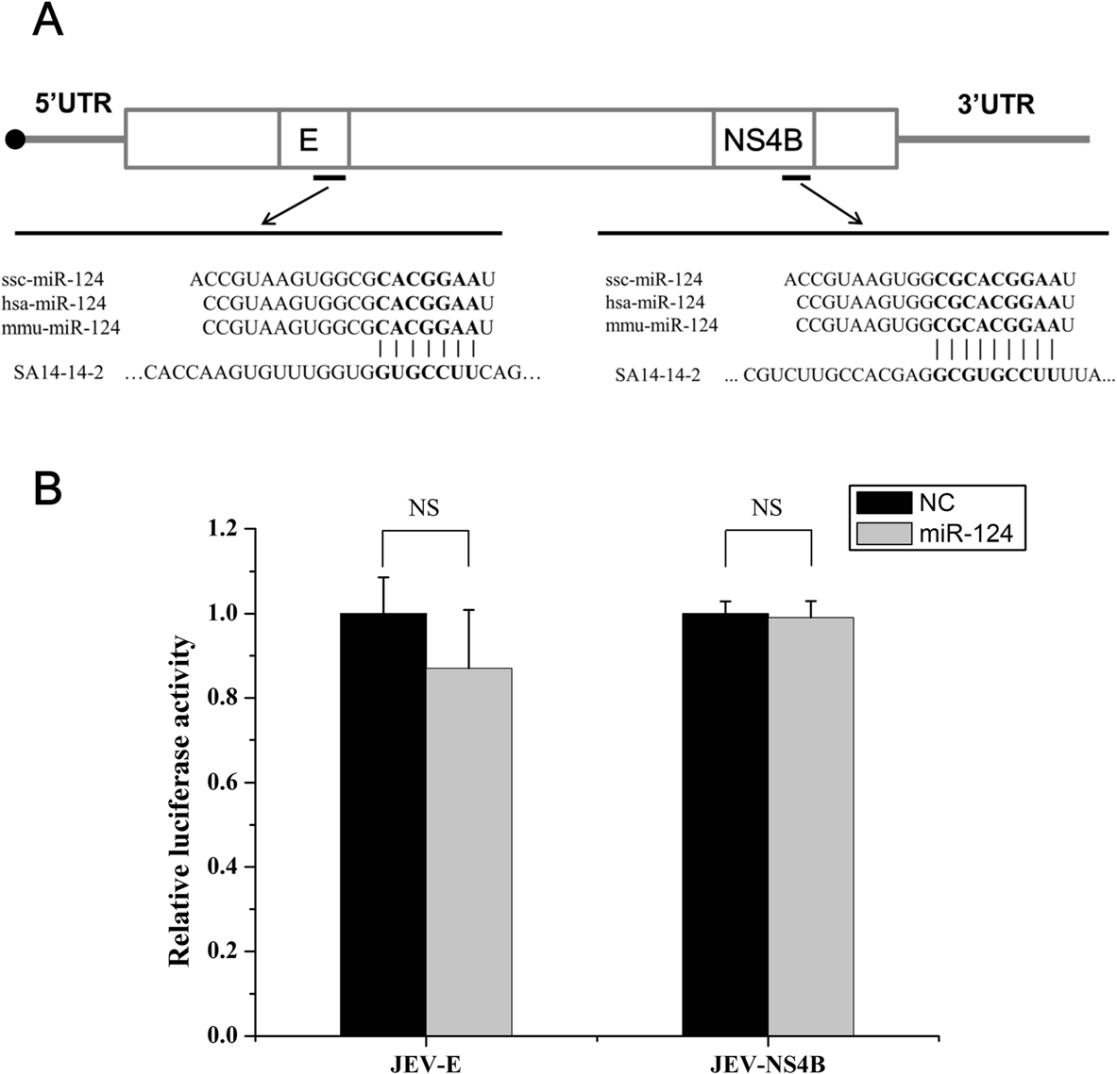
JEV genomic RNA is not the target of miR-124. **a** Computational prediction of potential miR-124 target sites in JEV genomic RNA. Sequence alignments of 10 representative JEV strains. **b** Effect of miR-124 mimic on the expression of luciferase from reporter constructs. Sequences of predicted target sites in the JEV genome were inserted into the dual luciferase reporter vector. BHK-21 cells were co-transfected with reporter constructs and the miR-124 or nonspecific (NC) miRNA mimics. The luciferase assay was performed 24 h after the transfection. Renilla luciferase values were normalized against firefly luciferase values. NS, not significant

We used ViTa software [36] to identify two potential miR-124 binding sites in the genomic RNA of the JEV strain. The two miR-124 binding sites are located in the E and NS4B genes (Fig. 2A).

These predictions were then validated by a luciferase reporter assay. Nucleotide sequences from JEV RNA predicted to interact with miR-124 were inserted into the 3’UTR of *h-luc* in the psiCHECK-2 vector. BHK-21 cells were co-transfected with the reporter construct and miR-124 mimic, and then assayed for luciferase activity at 24 h post-transfection (hpt). However, transfection of the miR-124 mimic did not significantly alter the expression of luciferase from the reporter constructs bearing either of the predicted miR-124 target sites from the JEV RNA (Fig. 2B). Therefore, the mechanism by which miR-124 inhibits virus replication is likely to involve miR-124-mediated regulation of host genes.

### DNM2 is a target of miR-124

**Fig. 3:**
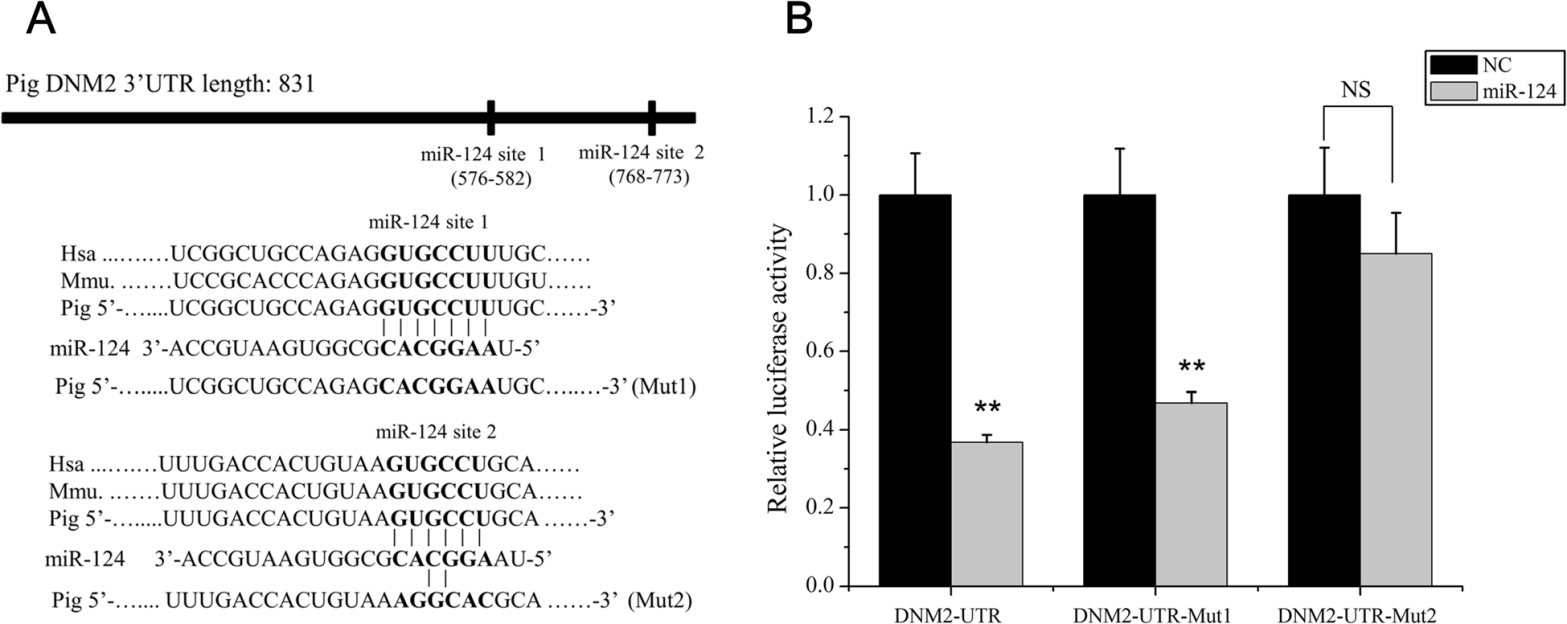
miR-124 interacts with the predicted target site in the 3’UTRs of DNM2 mRNA. **a** Potential base pairing is indicated by vertical lines between the sequence of miR-124 (bold) and its target sequences within the 3’ UTR of DNM2 in pig, human and mouse. The DNM2 3’UTR mutants (Mut1 and Mut2), containing a mutated miR-124 binding site, are shown. **b** BHK-21 cells were co-transfected with luciferase reporter vectors containing the wild type or mutant 3’UTR of porcine DNM2 and the miR-124 mimic or miR-negative control. The luciferase assay was performed 24 h after the transfection. Renilla luciferase values were normalized against firefly luciferase values, **p < 0.01, NS, not significant

Because miR-124 did not target the JEV RNA, we instead focused on exploring host factors that may be involved in these antiviral processes. To elucidate which host genes are targeted by miR-124, a genome-wide computational analysis of all potential miR-124 targets was performed using the TargetScan software (http://www.targetscan.org/) [37]. A cluster analysis further narrowed the number of predictions and showed that there were 28 genes (Additional file 1) involved in endocytosis pathways, including the DNM2 gene which plays a critical role in viral endocytosis. The two miR-124 binding sites in the 3’UTR of DNM2 were conserved in Vertebrata, which includes pigs, mice and humans. Interestingly, nucleotides 3–8 of the miR-124 (fall within seed region) was perfectly complementary with DNM 3’UTR in the miR-124 site 2, the other target position from the canonical seed region (nucleotides 2–8). (Fig. 3A).

**Fig. 4:**
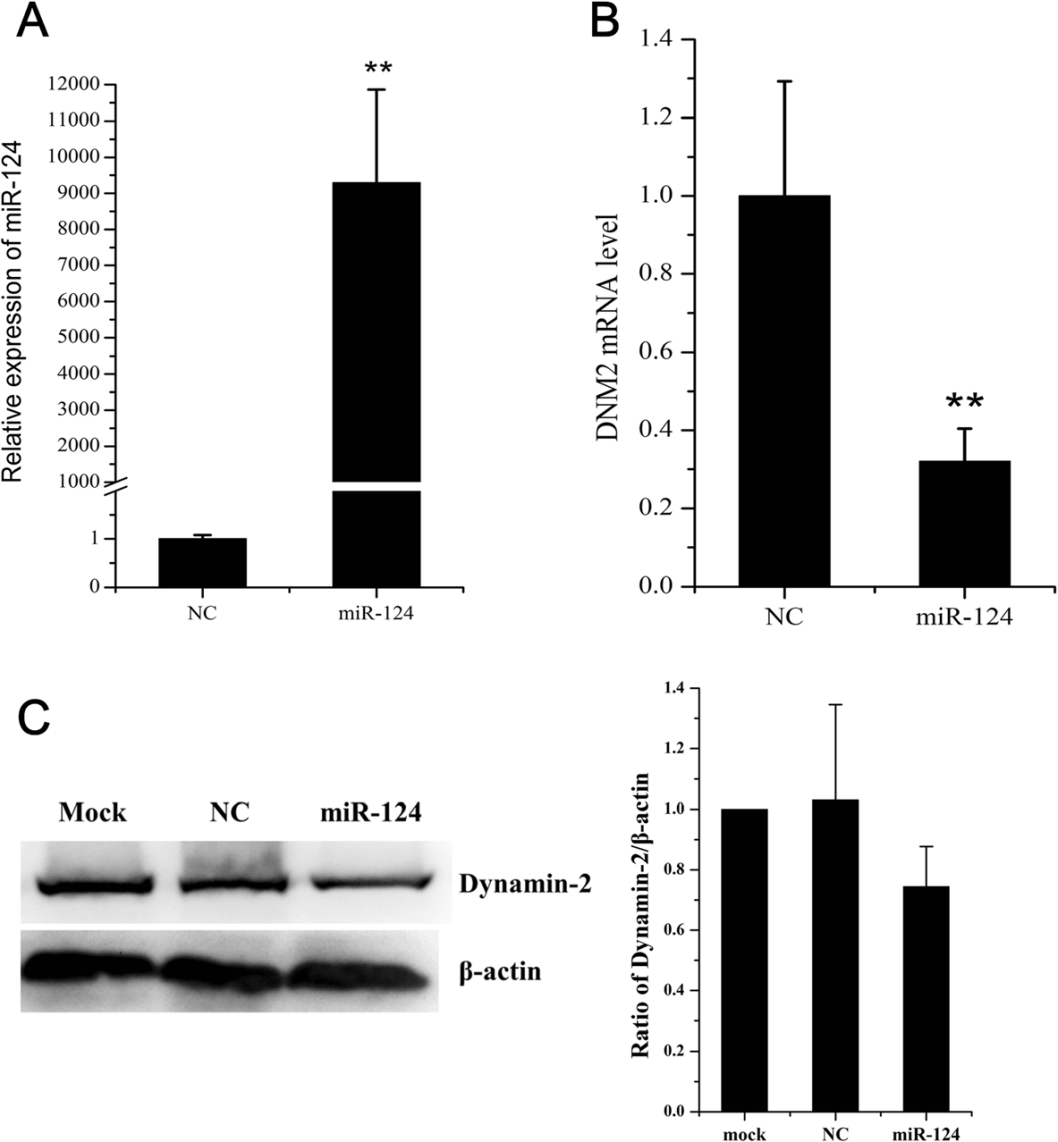
miR-124 suppresses porcine DNM2 expression. PK15 cells were transfected with nonspecific (NC) or miR-124 mimics as indicated. The expression level of miR-124 (**a**) and DNM2 mRNA (**b**) was assessed by qRT-PCR at 48 h after transfection. Expression levels of miR-124 and DNM2 were normalized to U6 snRNA and RPL32, respectively. **c** Western blot analysis of dynamin-2 expression in cells transfected with the NC and miR-124 mimics at 48 h after transfection. β-actin was used as a loading control, **p < 0.01

To confirm that miR-124 directly targets the 3’UTR of DNM2, dual-luciferase reporter plasmids (psiCHECK2 Vector) carrying the DNM2 3’UTR with the wild-type or base-pair mutant miR-124 binding regions was constructed (Fig. 3A). After co-transfection of BHK-21 cells with psiCHECK2-DNM2 3’UTR and miR-124 mimic, the luciferase activity was measured at 24 hpt. Luciferase activity markedly decreased when cells were co-transfected with the miR-124 mimic and wild-type or mutant target site1(Mut1) DNM2 3’UTR plasmids in comparison with NC mimic (Fig. 3B). However, the DNM2 3’UTR Mut2 reversed the inhibition of luciferase activity compared to DNM2 3’UTR wild-type (Fig. 3B). Therefore,

miR-124 targets pig DNM2 gene mainly interacting with the second target site (miR-124 site2) of DNM 3’UTR. In addition, we assessed the effect of miR-124 mimic transfections on porcine DNM2 mRNA and protein levels. PK15 cells were transfected with miR-124 specific or nonspecific miRNA mimics, and at 48 hpt, the expression of DNM2 was analyzed by qRT-PCR and western blot analysis. The expression of miR-124 was significantly increased in the miR-124 transfection group when compared to the NC mimic (Fig. 4A). Transfection of the miR-124 mimic resulted in a significant reduction in the mRNA and protein levels of the porcine DNM2 gene compared to cells transfected with the NC mimic (Fig. 4B, C).

### DNM2 is required for JEV replication

**Fig. 5:**
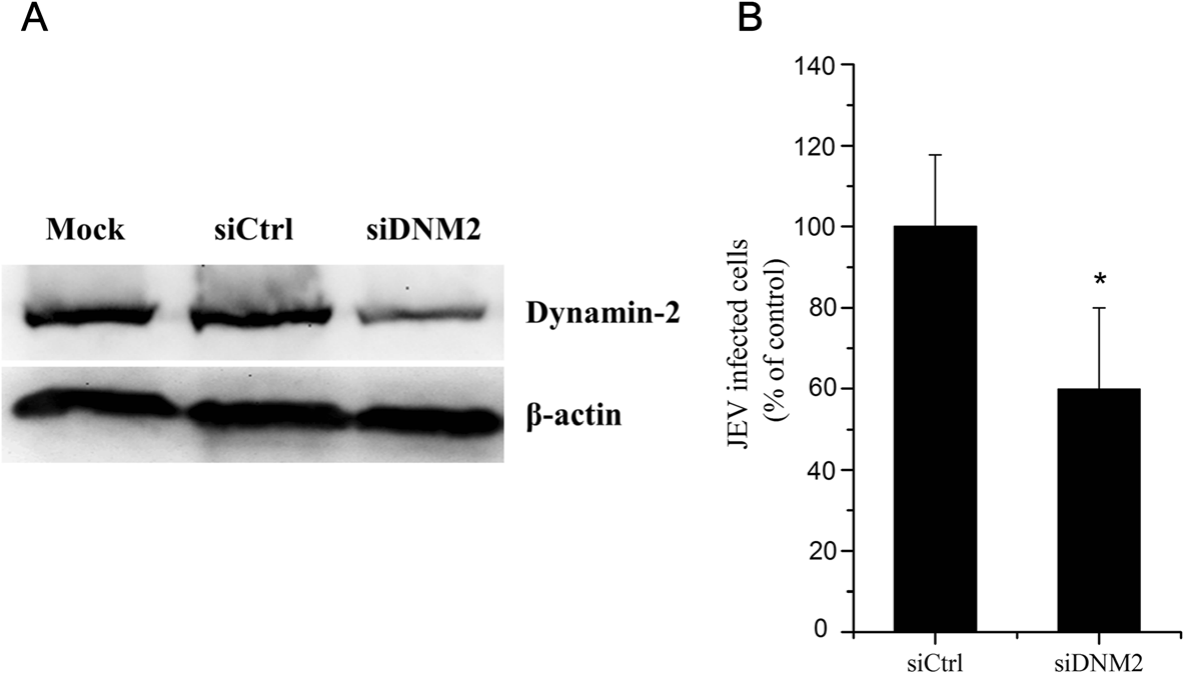
Effect of DNM2 depletion on JEV replication. PK15 cells were transfected with siRNA targeting DNM2 or non-targeting siRNA (siCtrl). **a** Dynamin-2 knockdown effect was analyzed at 48 h post-transfection by immunoblotting. **b** The transfected cells were infected with JEV at 48 h post-transfection and samples were collected at 36 hpi. The percentage of internalized viruses in the transfected cells was determined by flow cytometry and normalized to the value for the siCtrl, *p < 0.05

In order to elucidate the role of DNM2 in JEV infection, we assessed viral replication in cells depleted in DNM2 by siRNA. PK15 cells were transfected with siRNA specific to porcine DNM2 or with nonspecific siRNA. The efficiency of siRNA-mediated knockdown of DNM2 was first determined by western blotting at 48 hpt. DNM2 knockdown was highly efficient, resulting in a 70 % decrease in DNM2 expression (Fig. 5A). To determine the effect of DMN2 depletion on JEV infection, cells transfected with siRNA were infected with an MOI = 1 of JEV at 48 hpt. Samples of cells were collected at 36 h post-infection (hpi) and the positively infected cells were determined by flow cytometry. The DNM2 knockdown cells showed a significant reduction in JEV infection compared to cells transfected with the control siRNA (Fig. 5B). These results show that DNM2 is necessary for JEV replication.

### miR-124 and DNM2 are inversely affected by JEV infection

**Fig. 6:**
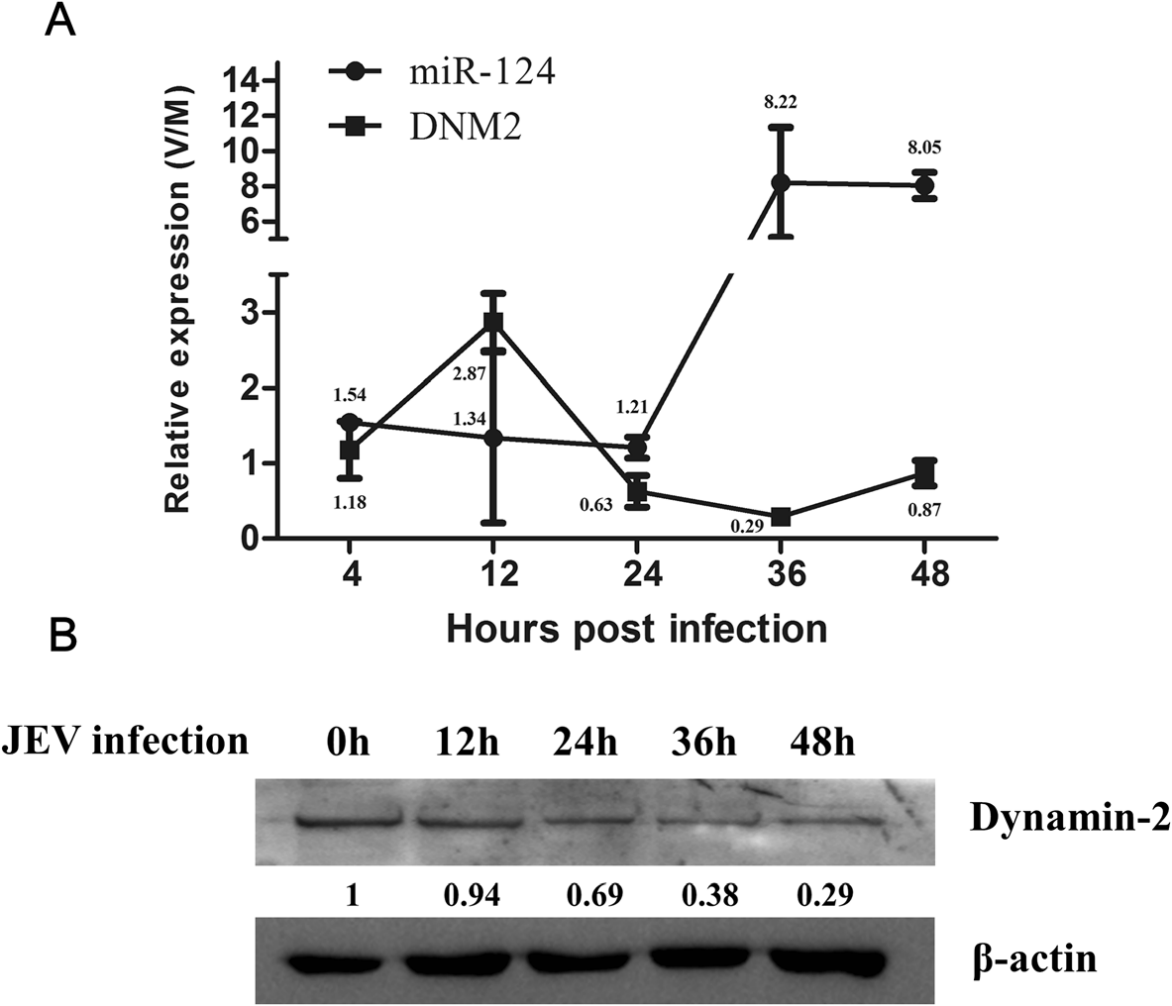
Expression of miR-124 and DNM2 in PK15 cells during JEV infection. **a** PK15 cells were infected with JEV. Mock-infected (M) or virus-infected (V) cells were harvested at the indicated times, and the miR-124 and DNM2 RNA levels were quantified using qRT-PCR. The data were normalized to the levels measured in mock-infected cells to provide relative expression levels (V/M). The results shown are means ± SD with triplicate data points. **b** Replicate cell cultures were evaluated by western blot for DNM2 protein expression. β-actin serves as a loading control

Previous studies indicated that expression of miR-124 is upregulated by JEV in swine testis cells [30]. DNM2 is required for the scission of nascent vesicles from parent membranes during endocytic events, which is believed to benefit JEV replication [38, 39]. To determine the levels of miR-124 and DNM2 during JEV infection, PK15 cells were infected with JEV, and the miR-124 and DNM2 mRNA levels were subsequently quantitated by qRT-PCR at different times post-infection. miR-124 levels increased as early as 4 hpi and showed modest increases of 1.2 to 1.5-fold in the first 24 hpi. At 36 and 48 hpi, miR-124 levels increased abruptly (8-fold) (Fig. 6A). In contrast, the DNM2 mRNA levels slightly increased as early as the first 12 hpi, and then declined gradually to 30 % of uninfected controls by 36 hpi (Fig. 6A). Dynamin-2 levels decreased after JEV infection at different time points (Fig. 6B). These results demonstrate that expression of miR-124 and DNM2 are differentially modulated by JEV infection, which indicates that miR-124 and DNM2 play different roles in influencing JEV replication.

## Discussion

MicroRNAs serve as multifunctional regulators, and the significance of miRNAs in virus-host interactions is becoming evident. A growing number of studies show that host miRNAs can drastically influence virus replication, either by facilitating enhanced replication [40] or by mediating the host antiviral response [17]. Therefore, many viruses, including JEV, modulate the expression of specific cellular miRNAs [22–25]. JEV is a significant pathogen of acute CNS inflammatory disease in humans. In addition, pigs act as amplifying hosts of JEV. However, the role of host miRNAs in JEV-host interactions and pathogenesis has not been extensively studied.

miR-124 is highly expressed in neurons, and it plays a notable role in neuronal differentiation [28]. In addition, miR-124 was attenuated in several tumors, and the overexpression of miR-124 inhibited the metastasis of breast cancer and hepatocellular carcinoma [41, 42]. Interestingly, previous studies showed that miR-124 could play an important role in virus entry by disturbing receptor-mediated endocytosis, and miR-124 was upregulated in JEV-infected porcine cells [30, 43]. These results suggest that miR-124 may be involved in the regulation of JEV infection. In order to determine the biological role of miR-124 in the host response to JEV infection, miR-124 was overexpressed through miRNA mimic transfection in order to assess its effect on JEV replication. The flow cytometry and Western blot experiments revealed that miR-124 exhibited a significant antiviral effect (Fig. 1). Thus, the key new finding of this study is that miR-124 is an anti-JEV miRNA.

miRNAs regulate many biological processes by interacting with their target genes. Host miRNAs were previously shown to directly target viral genomic RNAs; for example, miR-122 facilitates hepatitis C virus replication [44], while miR-130b and miR-181 suppress the porcine reproductive and respiratory syndrome virus (PRRSV) [19, 45]. According to the online software analysis, there were two potential miR-124 binding sites in the JEV genomic RNA. However, the luciferase reporter assay showed that miR-124 does not directly interact with the 2 predicted sites in the JEV RNA (Fig. 2). These data indicate that the antiviral effect of miR-124 is mediated by its interaction with the host genes. In order to test this hypothesis, we screened for miR-124 targets using TargetScan and ultimately focused on the DNM2 gene. The luciferase reporter assay showed that miR-124 directly interacts with the 3’ UTR of DNM2 and that DNM2 mRNA and protein levels were reduced in cells overexpressing miR-124 (Figs. 3 and 4).

Dynamin-2, a GTPase responsible for vesicle scission, forms helical polymers around the membrane neck of nascent endocytic buds and, upon GTP hydrolysis, mediates the fission of vesicles from the plasma membrane [46]. Thus, the research showed that other factors may assist with the action of dynamin-2 in vivo because dynamin-2 is sufficient to mediate membrane fission [47]. In addition, dynamin-2 was required for the caveolae and clathrin-mediated endocytosis pathways [47, 48]. We previously found that JEV infected PK15 cells via clathrin-dependent endocytosis [8], and miR-124 reduced caveolar density in porcine PK15 cells [43]. Therefore, dynamin-2 may play an important role in JEV infection. Indeed, siRNA knockdown was performed in order to assess its effect on JEV replication in DNM2 depleted cells. The knockdown experiment demonstrated that dynamin-2 was required for JEV replication (Fig. 5). However, the step at which the inhibition of JEV infection by dynamin-2 occurs, perhaps at entry, still needs to be demonstrated.

Previous studies using high-throughput sequencing technology found that miR-124 was downregulated by JEV in swine testis cells [30]. In this study, quantitative methods were used to determine expression levels of miR-124 in PK15 cells before and after JEV infection. The results confirmed the previous reports and further showed that miR-124 was upregulated immediately upon infection (4 hpi) and remained upregulated throughout the infection. In contrast, DNM2 expression levels increased during early JEV infection (first 12 hpi) and then sharply declined (24 hpi) in mRNA level, however, DNM2 protein level always remained downregulated throughout the infection (Fig. 6). Considering the important role of DNM2 in the regulation of virus entry, it is possible that miR-124-mediated suppression of DNM2 expression may block JEV-induced vesicle scission, thereby contributing to the antiviral effect of miR-124. However, miR-124 may also target other host regulators, and their roles in JEV replication may have yet to be evaluated.

## Conclusion

Taken together, this study demonstrates that overexpression of miR-124 inhibits JEV replication in PK15 cells. Subsequently, miR-124 suppresses expression of the DNM2 gene via targeting its 3’ UTR sequence. DNM2 is required for efficient JEV replication. In addition, the expression of miR-124 and DNM2 is inversely affected by JEV infection. This finding implies that the miR-124-DNM2-pathway plays an important role in the suppression of virus replication. These findings further highlight that mammalian miRNAs may have important implications for controlling virus infections.

## Methods

### Cell culture, transfection and viral infection

PK15 and baby hamster kidney (BHK-21) cell lines (obtained from the American Type Culture Collection, Manassas, VA) were grown in Dulbecco’s Modified Eagle Medium (DMEM, High glucose, Thermo Scientific Hyclone, Beijing, China) supplemented with 10 % fetal bovine serum (Gibco, Life Technologies, Austin, TX) and maintained in a humidified incubator at 37 °C and 5 % CO2.

The miRNA mimics and siRNA (siDNM2) were synthesized by GenePharma (Shanghai, China). The sequences of siDNM2 and mimics were as follows: siDNM2 5’-GGACAUGAUCCUGCAGUUTT-3’ [49], miR-124 mimics: 5’- UAAGGCACGCGGUGAAUGCCA-3’, NC (Negative control): 5’-UUCUCCGAACGUGUCACGUTT-3’. PK15 cells were seeded in 24- or 6-well plates and grown to approximately 50 % confluence for transfection. The cells were transfected with 50 pmol miRNA mimics or siDNM2 using Lipofectamine 2000 reagent (Invitrogen, Carlsbad, CA) according to the manufacturer’s protocol. The cells were cultured at the indicated times before harvesting.

The JEV attenuated strain SA14-14-2 (GenBank accession: AF315119.1) was propagated in BHK-21 cells as described previously [8]. All infections were carried out by incubating the cells with a small volume of medium (300 μL in per well of 6-well cell culture plate) containing virus at the MOI = 1, then the inoculum was removed, the cells were washed three times with Phosphate Buffered Saline (PBS, Thermo Scientific Hyclone, Beijing, China) and fresh media was added. Infections were performed and the infected cells were maintained in DMEM supplemented with 2 % FBS.

### RNA extraction, reverse transcription, and quantitative real-time PCR (qPCR)

Total RNA was isolated using TRIzol reagent according to the manufacturer’s instructions. Genomic DNA was removed from RNA samples by incubation with DNase I (Promega). The synthesis of cDNA was conducted with 1 μg of total RNA using an RT-PCR reagent kit (PrimeScript™ 1st Strand cDNA Synthesis Kit, TaKaRa Biotechnology, DaLian, China) with an oligo-dT primer for mRNA and a special stem-loop primer for miRNAs [50]. Real-time quantitative PCR was performed on an Stratagene Mx3000P instrument (Agilent), using SYBR Green detection chemistry (SYBR® Premix Ex Taq™ II, TaKaRa). Relative expression levels were calculated by applying the 2^-ΔΔCt^ method [51] using reference gene RPL32 for mRNA and U6 snRNA for miRNA relative to the control samples. The PCR program was: 95 °C for 2 min followed by 40 cycles of 95 °C for 10 s, 60°Cfor 20 s, and 72 °C for 15 s followed by melting curve analysis. The sequences of the qPCR primers are listed in Table 1.

**Table 1:** Primers used for reverse transcription, real-time quantification PCR

Name	Sequence (5’-3’)	TM (^o^C)	Size (bp)
miR-124-RT	CTCAACTGGTGTCGTGGAGTCGGCAA		
	TTCAGTTGAGTGGCATTC		
miR-124-S	TCGGCAGGTAAGGCACGCGGTG	62	64
miR-124-A	TCAACTGGTGTCGTGGAGTCGGC		
U6_S	GTGCTCGCTTCGGCAGCACATAT	62	106
U6_A	AAAATATGGAACGCTTCACGAA		
DNM2- S	CCCACTCGCAAGACCAAA	60	132
DNM2-A	GATGCGATTGATTCGGGC		
RPL32-S	CGGAAGTTTCTGGTACACAATGTAA	60	96
RPL32-A	TGGAAGAGACGTTGTGAGCAA		

S Sense primer, A anti-sense primer, RT loop primer for microRNA reverse transcript

### Protein extraction and western blot

Cells were harvested 48 h after transfection, and lysates were prepared using RIPA buffer (50 mM Tris (pH 7.4), 150 mM NaCl, 1 % Triton X-100, 1 % sodium deoxycholate, 0.1 % SDS, 1 mM phenylmethylsulfonyl fluoride [PMSF], Beyotime, China). The protein concentration was determined with the BCA Protein Assay kit (Solarbio, China). Equal amounts of protein lysate were separated on 12 % SDS-polyacrylamide gels and transferred to PVDF membranes (Millipore). The membranes were blocked with 5 % nonfat milk in Tris-buffered saline containing 0.1 % Tween-20 (TBST) and then incubated with primary antibodies specific for mouse anti-JEV E, goat anti-Dynamin-2 (sc-6400, Santa Cruz), or rabbit anti-ß-Actin (#4967,1:1000, Cell Signaling) overnight at 4 °C. Membranes were then washed three times and incubated with a horseradish peroxidase-conjugated secondary antibody (Proteintech Group, Wuhan, China) for 1 h at ambient temperature. Finally, protein bands were visualized by addition of the SuperSignal West Pico chemiluminescent substrate (Thermo, Rockford, IL), with β-actin as a control. The mean densities of the protein bands were measured by ImageJ software (National Institutes of Health, Bethesda, Maryland).

### Plasmid construction and luciferase reporter assay

Luciferase reporter plasmids were constructed by cloning double-stranded synthetic oligonucleotides (Table 2) containing miRNA target sequences flanked by XhoI and NotI sites into the XhoI/NotI digested psiCHECK-2 Dual-Luciferase Expression Vector (Promega, Madison, WI). All constructs were verified by sequencing using the universal primers listed in Table 2. BHK-21 cells were co-transfected with 200 ng of the psiCHECK2 constructs and 30 nM of the miR-124 mimic or miR-negative control in each well of a 24-well plate. Twenty-four hours after transfection, firefly and Renilla luciferase activities were measured using the Dual-Glo Luciferase assay system (Promega) according to the manufacturer’s instructions. Normalized data were calculated as the quotient of Renilla/firefly luciferase activities. Three independent experiments were performed in triplicate.

**Table 2:** Primers used for luciferase reporter gene vector construct

Name	Sequence (5’-3’)	TM (^o^C)	Size (bp)
DNM2-3UTR-S	CA **CTCGAG**AGAAACAGCACCAAGGGAG	62	412
DNM2-3UTR-A	TA **GCGGCCGC**CAGGCACTTACAGTGGTCAA		
DNM2-3UTR-Mut1-A	GGACCTGGCATTCCGTGCTCTGGCAGCCGAGGCTG		
DNM2-3UTR-Mut1-S	GCTGCCAGAGCACGGAATGCCAGGTCCGGAAAACC		
DNM2-3UTR-Mut2-A	TAGCGGCCGCCGTGCCTTTACAGTGGTCAAACCGA		
JEV-E-S	CA **CTCGAG**AACTCAAAGGTGCTGGTC	62	387
JEV-E-A	TA **GCGGCCGC**ACCTCCTGTGGCTAAGAA		
JEV-NS4B-S	CA **CTCGAG**GTCTTGACCGTGGTTGGA	60	378
JEV-NS4B-A	TA **GCGGCCGC**CGTTGTGAGGGTGACTTG		
Universal primer-S	CCACTTCAGCCAGGAGGA	60	216
Universal primer-A	GGTCCGAAGACTCATTTAGA		

S Sense primer, A anti-sense primer, the underline indicates additional bases and restriction enzyme recognition sites are bold

### Flow cytometry

PK15 cells infected with JEV were washed one time with PBS, detached and transferred to 1.5 ml centrifuge tubes. The cells were centrifuged at 1000 rpm/min for 10 min and fixed with 4 % paraformaldehyde (Solarbio, Beijing, China) for 15 min at room temperature. After being permeabilized with Triton X-100(amresco, Solon, OH), the cells were incubated with mouse anti-JEV E antibody overnight at 4 °C. The cells were washed 3 times with PBS then incubated with Alexa Fluor 488 goat anti-mouse IgG (Invitrogen) at 1:200 for 1 h at room temperature and protected from light. The cells were washed with PBS, resuspended in 500 μl PBS and analyzed using a FACScan flow cytometer with CellQuest pro software (BD Biosciences, San Jose, CA). The cells were counted as infected if their fluorescence density was greater than the intensity of the uninfected cells. The amount of infected cells relative to the untreated or siCtrl-transfected controls was given as percent infection. At least 10,000 cells were analyzed per sample. Three independent experiments were performed in triplicate.

### Statistical analysis

The results were presented as the mean ± standard deviation (SD). Statistical significance was assessed by Student’s *t*-test, and statistical significance was ascribed when *p < 0.05, **p < 0.01.

## Additional file

Additional file 1: The pathways of predicted target genes of miR-124. (XLSX 19 kb)
